# Chemical Composition and Antimicrobial Potential of Palm Leaf Extracts from Babaçu (*Attalea speciosa*), Buriti (*Mauritia flexuosa*), and Macaúba (*Acrocomia aculeata*)

**DOI:** 10.1155/2016/9734181

**Published:** 2016-07-26

**Authors:** Adriana Idalina Torcato de Oliveira, Talal Suleiman Mahmoud, Guilherme Nobre L. do Nascimento, Juliana Fonseca Moreira da Silva, Raphael Sanzio Pimenta, Paula Benevides de Morais

**Affiliations:** ^1^Laboratório de Microbiologia Ambiental e Biotecnologia (LAMBIO), Universidade Federal do Tocantins, 77001-923 Palmas, TO, Brazil; ^2^Centro de Estudos do Mar (CEM), Federal University of Paraná, 83255-976 Pontal do Paraná, PR, Brazil; ^3^Laboratory of Basic and Health Sciences, Federal University of Tocantins, 77001-923 Palmas, TO, Brazil

## Abstract

Babaçu (*A. speciosa*), Buriti (*M. flexuosa*), and Macaúba (*A. aculeata*) are palm trees typical of the ecotone area between Cerrado and the Amazon rainforest. The purpose of this study was to evaluate the antimicrobial potential of the extracts prepared from the leaves of those palms as well as determine their chemical compositions. The ethanol extracts were prepared in a Soxhlet apparatus and tested by disk diffusion and agar dilution technique against* Staphylococcus aureus*,* Enterococcus faecalis*,* Escherichia coli, Pseudomonas aeruginosa*,* Candida albicans*, and* Candida parapsilosis.* However, there was no significant activity at concentrations of 25, 50, and 100 mg·Ml^−1^. Moreover, the phytochemical analysis revealed the presence of tannins, flavonoids, catechins, steroids, triterpenes, and saponins. Gas chromatography (GC/MS) analysis also identified organic acids, such as capric (decanoic) acid, lauric (dodecanoic) acid, myristic (tetradecanoic) acid, phthalic (1,2-benzenedicarboxylic) acid, palmitic (hexadecanoic) acid, stearic (octadecanoic) acid, linoleic (9,12-octadecadienoic) acid (omega-6), linolenic (octadecatrienoic) acid (omega-3), and the terpenes citronellol and phytol. Based on the chemical composition in the palm leaf extracts, the palms have the potential to be useful in the food, cosmetic, and pharmaceutical industries.

## 1. Introduction

**Figure 1:**
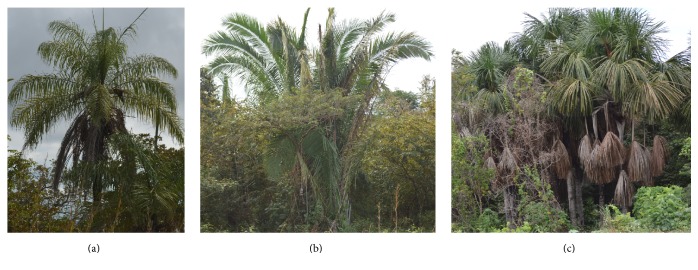
Photos personnel file. (a)* Acrocomia aculeata*. (b)* Attalea speciosa*. (c)* Mauritia flexuosa*.

Brazil owns 20% of all the biodiversity in the world [1]. Unfortunately, only 10% of all plant species have been included in chemical or biological studies [2]. Generally, the therapeutic use of plants is known by conventional wisdom. However, this use should be based not only on observation but also on the results of scientific experimentation [3]. The pharmacological activity of a plant is attributable to one or more active chemical substances found in the plant tissue [4]. The phanerogams produce chemical compounds via primary and secondary metabolism. Secondary metabolites are compounds that play an important role in plant survival, providing a defense mechanism against predation by insects, herbivores, and microorganisms [5]. The Arecaceae family includes several important tropical plants, especially palm trees. Many authors consider the Arecaceae family of plants to be the most important in the life of forest people [6]. In addition, biodiversity of the palm flora of Brazil is quite rich, with an estimated 221 species [7] and 39 genera, the majority of which are found in the Amazon forest. Furthermore, in Tocantins state, it is possible to find several species of palms, including* Acrocomia aculeata* (Jacq.) Lodd. ex Mart. (Macaúba),* Attalea speciosa* Mart. ex Spreng. (Babaçu),* Mauritia flexuosa* L.f. (Buriti), and others [8] (Figure 1). Given the lack of scientific research on these palm species, this work aims to study the chemical properties and evaluate antimicrobial activity related to the ethanolic extracts obtained from their leaves.

## 2. Materials and Methods

### 2.1. Sample Preparation and Extraction Using Solvent

Plant samples from the palm trees* A. speciosa, M. flexuosa*, and* A. Aculeata* were made in April 2015 at Escola de Medicina Veterinária e Zootecnia, EMVZ Campus (7°06′46, 8′′S48°11′34, 6′′W) of the Universidade Federal do Tocantins (UFT). Control species were located in the Herbarium HTO of UFT with the following registry numbers:* Attalea speciosa* (10.953),* Mauritia flexuosa* (10.952), and* Acrocomia aculeata* (10.954). In addition, dry material of the palm trees was obtained from green leaves that were cut using common scissors and dried in an oven (FANEM, São Paulo, Brazil) at 45–48°C for 6 hours. The moisture content of the samples was determined based on the methods of Institute Adolf Lutz [9]. The percent humidity (*U*%) for each species was calculated according to the following formula:

(1) $$\begin{array}{c} U\%=\left[\;\frac{\left(\text{Green material}-\text{dry material}\right)}{\text{green material}}\;\right]*\text{100}. \end{array}$$

The extraction of the chemical compounds was performed using a Soxhlet extractor [10]. The dry material (leaves) was weighed directly in cellulose thimbles (Babaçu: 8.595 g, Buriti: 7.050 g, Macaúba: 10.004 g) and then was loaded into the Soxhlet. All extractions used 250 mL of ethanol (Sigma-Aldrich, Rio de Janeiro, Brazil) as the solvent, and the extraction was carried out over 5 hours with the water cooling system set to 18°C. Ethanol is a solvent capable of extraction of a wider group of both polar and apolar compounds such as organic acids, essential oils, lipids, and pigments. It also presents a low toxicity being considered a less aggressive solvent. After extraction, the solvent was removed by rotary evaporation (CIENLAB, São Paulo, Brazil). The yield (*R*%) of each extract was calculated based on the amount of dry matter according to the following equation:

(2) $$\begin{array}{c} R\%=\left[\;\frac{\text{Mo}}{\text{Bm}}-\frac{\left(\text{Bm}\cdot U\right)}{100}\;\right]\cdot \text{100} \end{array}$$

in which Mo is the mass of extract (g), Bm is the aerial biomass (g), *U* is the humidity, and 100 is the conversion factor for a percentage.

### 2.2. Phytochemical Screening

The phytochemical screening of extracts was performed in triplicate to identify secondary metabolites, such as tannins, flavonoids, catechins, carotenoids, organic acids, cardioactive glycosides, steroids and triterpenoids, saponins, sesquiterpene and other lactones, azulenes, coumarins, alkaloids, and anthraquinones [11].

### 2.3. Antimicrobial Activity

To evaluate the antimicrobial activity of the extracts, we used standard strains (American Type Collection Culture (ATCC)) that were obtained from the Oswaldo Cruz Foundation (Fiocruz, Rio de Janeiro, Brazil). The Gram-positive bacteria* Staphylococcus aureus* (ATCC 6538) and* Enterococcus faecalis* (ATCC 4083) and the Gram-negative bacteria* Escherichia coli* (ATCC 25922) and* Pseudomonas aeruginosa* (ATCC 27853) that are extensively used for antimicrobial tests of plant compounds were used. Additionally,* Candida albicans* (access number 4006) and* Candida parapsilosis* (access number 40038), which are leveduriformes fungi, were included in the test. The methodology was based on the disk diffusion method of Kirby-Bauer and the procedure was performed following the Performance Standards for Antimicrobial Disk Susceptibility Test [12]. The extracts were diluted in a mixture with dimethyl sulfoxide (DMSO) 10% (Sigma-Aldrich, Rio de Janeiro, Brazil), Tween-80 emulsifier 0.02% (Synth, São Paulo, Brazil), and saline solution 0.9% [13]. The concentrations of the final solutions for each extract were 100.0 mg·mL^−1^, 50.0 mg·mL^−1^, and 25.0 mg·mL^−1^. Disks treated with 10% DMSO were used as the negative control, while the positive control disks were treated with gentamicin (10 *μ*g/disk), chloramphenicol (30 *μ*g/disk), or fluconazole (30 *μ*g/disk). Müller-Hinton agar (bacteria) and Sabouraud Dextrose Agar (fungi) were used as growth media.

### 2.4. GC/MS

The chemical compounds in the plant extracts were derivatized (transesterification reaction) through acid catalysis of boron trifluoride in methanol with heat conditions according to Meher et al. (2006) [14]. The analyses were performed using a Shimadzu type GC/MS QP, 2010 Plus Model, which has a capillary column of fused silica HP-5MS (30 m × 0.25 mm × 0.25 *μ*m). The heating was ramped between 60 and 240°C at rate of 3°C/min. The injector temperature was 250°C in the splitless mode, and helium gas was used at a speed of 1.2 mL·min⁡^−1^. The electron energy was 70 eV, and the temperature of the ion source was 200°C. Finally, the identification of compounds was made by comparison of the peak mass data with the data in the NIST-08 (National Institute of Standards and Technology) library.

## 3. Results and Discussion

**Figure 2:**
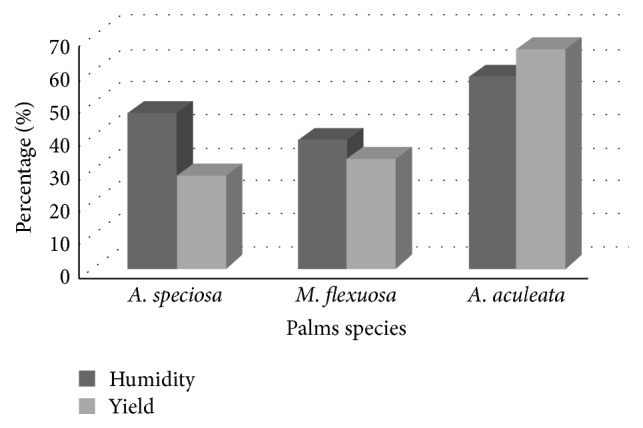
Humidity and yield extraction for* A. speciosa*,* M. flexuosa*, and* A. aculeata*.

The humidity percentage of the palm trees was 47.23% for Babaçu, 38.79% for Buriti, and 57.93% for Macaúba. These high humidity percentages are attributable to harvest during the rainy season. The extraction method using a Soxhlet extractor [15] resulted in a yield of 28.19% for* A. speciosa*, 33.14% for* M. flexuosa*, and 66.39% for* A. aculeata *shown in Figure 2. This is probably due to the part of the leaves used, since the extraction was made from* A. aculeata* folioles whereas in* A. speciosa* and* M. flexuosa* the whole leaf was used which included the blade with midrib and the petiole. This resulted in a drier and more powdery substrate of* A. aculeata* for the solvent to work.

The phytochemical screening of the leaf extracts from* M. flexuosa* and* A. aculeata* revealed the presence of tannins, flavonoids, catechins, steroids and/or triterpenoids, and saponins. However, the extract from* A. speciosa* revealed only flavonoids, steroids and/or triterpenoids, and saponins. These results indicate that the leaves of the palm trees that were studied hold promise for scientific use due to their secondary metabolites, which have biological and pharmacological activities that were found in the analysis.

The antimicrobial activity of the chemical compound found in the leaves of* A. speciosa*,* M. flexuosa*, and* A. aculeata* was tested against four bacterial strains and two strains of leveduriformes fungi; both are pathogens for humans and showed no sensitivity to the extracts. While the positive controls showed the expected zones of inhibition, there was no significant antimicrobial activity against the tested microorganisms based on the agar-diffusion results. The selection of microorganisms was made to verify the antimicrobial activity of the extracts. However, further tests using other strains, including plant pathogens, or the use of alternative methods should be considered.

Gas chromatography analysis of* A. speciosa*,* M. flexuosa*, and* A. aculeata* leaf extracts showed ten (10) chemical compounds (Table 1) that are known to have biological and pharmacological properties.

The identified compounds represent a mixture of esters derived from saturated fatty acids, unsaturated fatty acids, aromatics, and terpenes. The extract of* M. flexuosa* showed the highest percentage of saturated fatty acids, which are responsible for food palatability. Palmitic acid and hexadecanoic acid were found in higher concentrations in* M. flexuosa* (20.35%) and* A. aculeata* (12.12%) extracts. These fatty acids are particularly useful for improving the textural properties of foods and are used in the cosmetic industry. Moreover, the linolenic fatty acid (octadecatrienoic acid) and linoleic acid (9,12-octadecadienoic acid) are the most important finding because they are essential fatty acids (EFAs). The three analyzed palm trees showed a ratio of linolenic/linoleic acid between 4 : 1 and 5 : 1, which is the most recommended for human nutrition by leading regulatory agencies in the world, including the Scientific Review Committee (SRC) and the World Health Organization (WHO) [16]. The phytol (3,7,11,15-tetramethyl-2-hexadecen-1-ol) was found at a low concentration in both* M. flexuosa* (6.28%) and* A. aculeata* (2.44%) extracts and is a component of the chlorophyll molecule, which is present in green leaves of various medicinal plants and used by the cosmetic industry. The natural acyclic monoterpene citronellol is a GRAS substance (Generally Recognized as Safe for food use) and has been found in several plants reported to have antifungal, antibacterial, antispasmodic, and hypotensive properties [17]. For this reason, the presence of terpenes revealed in the phytochemical analysis were confirmed.

## 4. Conclusion

The use of ethanol has proved to be very favorable due to its low cost, its ability to be obtained by biotechnological processes, and its low toxicity [18, 19]. Although phytochemical tests did not reveal the presence of organic acids, they were verified by gas chromatography, which is a more precise method, especially when a compound is present in low concentrations. Considering this, the chemical composition of the palm tree leaves that were studied requires special consideration and attention in their interpretation. While they may vary due to environmental and/or genetic factors, this study contributes to the knowledge of the species and the expansion of its application in biotechnology. In summary, the results obtained contribute to a better understanding of the relationship between the chemical composition present in the leaves of palm trees and their scientific potential.

## Figures and Tables

**Table 1:** The major chemical compounds detected (area, %) and retention time (RT) in the leaf extracts of *A. speciosa*,* M. flexuosa*, and *A. aculeata* by GC/MS analysis.

Compounds	*A. speciosa*		*M. flexuosa*		*A. aculeata*	
	RT (min)	Area%	RT (min)	Area%	RT (min)	Area%
Capric acid, C_11:0_	20.416	2.26	20.429	2.64	nd	—
Lauric acid, C_12:0_	nd	—	28.925	1.29	nd	—
Myristic acid, C_14:0_	nd	—	36.727	1.28	nd	—
Phthalic acid, C_6_H_4_	nd	—	41.883	1.54	41.866	1.29
Palmitic acid, C_16:0_	43.927	3.27	43.861	20.35	43.816	12.12
Phytol, C_20_H_40_O	nd	—	47.515	6.28	47.483	2.44
Citronellol, C_10_H_20_O	48.408	7.65	48.437	11.75	48.401	7.63
Linoleic acid, C_18:2_ (*ω*6)	49.282	4.62	49.317	5.61	49.280	3.84
Linolenic acid, C_18:3_ (*ω*3)	49.515	20.65	49.540	19.94	49.501	18.92
Stearic acid, C_18:0_	50.291	1.93	50.320	3.82	50.291	2.78

RT: retention time in minutes; area: proportional peak area; nd: not detected.
